# Autoantibodies to Tailor-Made Panels of Tumor-Associated Antigens in Breast Carcinoma

**DOI:** 10.1155/2011/982425

**Published:** 2011-03-03

**Authors:** Ettie Piura, Benjamin Piura

**Affiliations:** ^1^Department of Obstetrics and Gynecology, Sapir Medical Center, Sackler School of Medicine, University of Tel-Aviv, Kfar-Saba 44281, Israel; ^2^Unit of Gynecologic Oncology, Department of Obstetrics and Gynecology, Soroka Medical Center and Faculty of Health Sciences, Ben-Gurion University of the Negev, Beer-Sheva 84101, Israel

## Abstract

Autoantibodies (AAbs) to tumor-associated antigens (TAAs) have been identified in the sera of cancer patients. In a previous review published in this journal, we have focused on recent knowledge related to circulating AAbs to individual TAAs in breast carcinoma. This review will focus on recent knowledge related to AAb assays to tailor-made panels of TAAs in breast carcinoma. So far, AAb assays to the following tailor-made panels of TAAs have been assessed in breast carcinoma: (1) p53, c-myc, HER2, NY-ESO-1, BRCA2, and MUC1, (2) IMP1, p62, Koc, p53, c-MYC, cyclin B1, and survivin, (3) PPIA, PRDX2, FKBP52, HSP-60, and MUC1, (4) MUC1, HER2, p53, and IGFBP2, (5) p53, HER2, IGFBP-2, and TOPO2*α*, (6) survivin and livin, (7) ASB-9, SERAC1, and RELT, and (8) p16, p53, and c-myc. Assessment of serum AAbs to a tailor-made panel of TAAs provides better sensitivity to diagnosis of breast carcinoma than measuring serum AAbs to a single TAA. Nevertheless, measurement of serum AAbs to a panel of TAAs for screening and early diagnosis of breast carcinoma is still investigational and should be carried out along with traditional diagnostic studies.

## 1. Introduction

The development of circulating autoantibodies (AAbs) to tumor-associated antigens (TAAs) has been observed to be associated with cancer [1, 2]. Unlike traditional tumor markers (e.g., CA-15-3, CA-19-9, CA-125, and CEA), which are soluble proteins shed by bulky tumors, serum AAbs to TAAs are detectable even before the tumor is overt clinically [2]. Indeed, Chapman et al. [3] reported and presented data from breast carcinoma patients that confirmed that AAbs to TAAs can be measured up to four years before mammography imaged the tumor. These striking data imply that the human immune system detects the TAAs as “nonself” and makes a humoral immune response very early in the disease process.

The number of women newly diagnosed with breast carcinoma in the USA during 2010 has been estimated to be 207,090 with an age-adjusted incidence of 121.8 per 100,000 women [4]. The number of women dead of breast carcinoma in the USA during 2010 has been estimated to be 39,840 with a death rate of 13.1 per 100,000 women [4]. In the USA, breast carcinoma is the first most common cancer among women (28% of all cancers in women) and the second most common cause of death from cancer, after lung carcinoma, in women (15% of all cancer deaths in women) [4]. The National Cancer Institute (NCI) has estimated that 12.2% (1/8) of women born today in the USA will be diagnosed with breast carcinoma at some time in their lives [5]. Nevertheless, the five-year survival rate overall of women with breast carcinoma in the USA is about 90% [6].

Worldwide, breast carcinoma is by far the most frequent cancer among women with an estimated 1.38 million new cases diagnosed in 2008 (23% of all malignancies in women) and ranks second overall (10.9%), after lung carcinoma, of all malignancies in both sexes [7]. The estimated incidence of breast carcinoma in 2008 worldwide has been 39 new cases per 100,000 women. The incidence has been estimated to vary from 19.3 in Eastern Africa to 89.9 in Western Europe, and is high (>80) in developed countries (except Japan) and low (<40) in most of the developing countries [7]. Breast carcinoma has been estimated to cause 458,000 deaths in 2008 worldwide (13.7% of all cancer deaths in women and 6% of all cancer deaths in both sexes). The estimated mortality from breast carcinoma in 2008 worldwide has been 12.5 deaths per 100,000 women. Breast carcinoma is the most frequent cause of death from cancer in women worldwide and the fifth cause of death from cancer, after lung, stomach, liver, and colorectal carcinoma, in both sexes [7].

Current screening modalities for breast carcinoma diagnosis include mammography, ultrasound (US), and magnetic resonance imaging (MRI); however, there is still an urgent need to develop an alternative modality of screening for earlier diagnosis [8]. The use of serum-soluble tumor antigens, such as CA-15-3 glycoprotein, as biomarkers for detection of breast carcinoma has been limited by their insufficient specificity and sensitivity, particularly for organ confined early-stage disease. Consequently, CA-15-3 is not recommended for use in the screening or detection of breast carcinoma [9–12]. This is in part due to the elevation of CA-15-3 in benign conditions including breast, liver, and kidney disorders and other cancers [13]. Thus, there is a need to discover novel biomarkers, such as serum AAbs to specific breast carcinoma TAAs, for screening, early diagnosis, prediction of prognosis, and monitoring of treatment. Thus far, only few circulating AAbs to specific breast carcinoma TAAs have been identified and investigated [14]. In breast carcinoma, like in other malignancies, the use of tailor-made panel of TAAs, rather than individual TAAs, enhances the likelihood of detecting cancer-associated AAbs with potential diagnostic value.

In a previous review published in the *Journal of Oncology*, we have focused on recent knowledge related to circulating AAbs to individual TAAs in breast carcinoma [14]. This review will focus on recent knowledge related to AAb assays to tailor-made panels of TAAs in breast carcinoma.  Table 1 summarizes the results of AAb assays to tailor-made panels of TAAs in breast carcinoma.

## 2. Autoantibody Assay to a Panel of Tumor-Associated Antigens Comprised of p53, c-myc, HER2, NY-ESO-1, BRCA2, and MUC1

Chapman et al. [3] examined by enzyme-linked immunosorbent assay (ELISA) the presence of AAbs to each of the following seven TAAs: p53, c-myc, HER2, NY-ESO-1, BRCA1, BRCA2, and MUC1 in the sera of 97 breast carcinoma patients, 40 ductal carcinoma *in situ* patients, and 94 normal controls. Positive seroreactivity was defined as an absorbance value greater than the mean plus 2 standard deviations of the normal cohort. The frequency of AAbs to individual antigens ranged from 8% to 34% in breast carcinoma and 3% to 23% in ductal carcinoma *in situ*. This corresponded to a significantly higher frequency of AAbs to each of the following six TAAs: p53, c-myc, HER2, NY-ESO-1, BRCA2, and MUC1, in breast carcinoma patients compared to controls and in ductal carcinoma *in situ* patients compared to controls. There was no significant difference, however, in the presence of AAbs to BRCA1 between breast carcinoma patients and controls, and between ductal carcinoma *in situ* patients and controls. Using an AAb assay to a panel comprised of the six TAAs, that each of them had elicited a significant higher rate of positive seroreactivity compared to controls (p53, c-myc, HER2, NY-ESO-1, BRCA2, and MUC1), revealed that AAbs to at least one of the six antigens were detected in the sera of 62/97 breast carcinoma patients and 18/40 ductal carcinoma *in situ* patients [3]. This corresponded to a sensitivity of 64% for breast carcinoma and a sensitivity of 45% for ductal carcinoma *in situ*. The specificity of AAb assay to individual antigens ranged from 91% to 98% for both breast carcinoma and ductal carcinoma *in situ*. The specificity of the AAb assay to the panel comprised of the above six TAAs was 85% for both breast carcinoma and ductal carcinoma *in situ* patients [3]. It has, thus, been shown that measuring AAbs to a tailor-made panel of TAAs provides better sensitivity than measuring AAbs to a single TAA. It has been suggested that AAb assay to a tailor-made panel of TAAs could be used as an aid to mammography in the detection of early breast carcinoma, especially in younger women at increased risk of breast carcinoma where mammography is known to have reduced sensitivity and specificity [3]. Moreover, with use of prototype assays for three TAAs, p53, c-myc, and MUC1, 6/9 (67%) women were reported to show AAbs between 7 and 27 months before the cancer was diagnosed on the screening mammograms. Further followup has confirmed that 9/15 (60%) patients in a larger cohort showed AAbs detectable up to 4 years before mammographic detection [3].

## 3. Autoantibody Assay to a Panel of Tumor-Associated Antigens Comprised of IMP1, p62, Koc, p53, c-MYC, Cyclin B1, and Survivin

Zhang et al. [15] examined by ELISA multiplex system the presence of AAbs to seven TAAs, IMP1, p62, Koc, p53, c-MYC, cyclin B1, and survivin, in the sera of 527 cancer patients (64, breast carcinoma; 45, colorectal carcinoma; 91, gastric carcinoma; 65, hepatocellular carcinoma; 56, lung carcinoma; and 206, prostate carcinoma) and 346 normal subjects. Positive seroreactivity was defined as an absorbance value above the mean plus 3 standard deviations of the normal cohort. In all types of cancer tested, although antibody frequencies to any individual TAA were variable and rarely exceeded 15%–20%, the successive addition of TAAs to the array to a total of seven antigens was associated with a stepwise increase of positive AAb reactions up to a range of 44%–68% [15]. In the sera of 64 patients with breast carcinoma, the successive addition of TAAs to the array to a total of seven antigens was associated with a stepwise increase of positive AAb reactions up to 43.8% [15]. It has been concluded that detection of AAbs in cancer can be enhanced by using a miniarray of several TAAs as target antigens [15, 21].

Koziol et al. [16] have analyzed the data of Zhang et al. [15] by classification trees based on the statistical method of recursive partitioning [22, 23]. By this method, Koziol et al. [16] have demonstrated that AAb assay to the tailor-made panel of the seven TAAs used by Zhang et al. [15] can discriminate between cancer patients and normal controls with reasonably high sensitivity and specificity, both typically exceeding 0.80. In breast carcinoma, using normal mean + 3 SDs as cutoff for positivity yielded a sensitivity of 70% and specificity of 95%, whereas using normal mean + 2 SDs as cutoff for positivity yielded a sensitivity of 92% and specificity of 85% [16]. The authors [16] concluded that classification trees based on a panel of seven TAAs can discriminate between breast carcinoma patients and normal subjects with reasonably high sensitivity and specificity. The multivariate approach of recursive partitioning using AAb assay to a tailor-made panel of TAAs yielded far higher values of sensitivity and specificity than an AAb assay to individual TAAs [16]. It has been concluded that multiple antigen miniarrays can provide accurate and valuable tools for cancer detection and diagnosis. Performance of the miniarrays might be enhanced by other combinations of TAAs appropriately selected for different cancer cohorts [16, 21].

## 4. Autoantibody Assay to a Panel of Tumor-Associated Antigens Comprised of PPIA, PRDX2, FKBP52, HSP-60, and MUC1

Desmetz et al. [17] examined the feasibility of using an AAb assay to a panel of TAAs as a method for early detection of breast carcinoma and, more particularly, carcinoma *in situ*. The authors examined by ELISA the presence of AAbs to five individual TAAs, PPIA, PRDX2, FKBP52, HSP-60, and MUC1, in sera from 60 breast carcinoma patients, 82 breast carcinoma *in situ* patients, and 93 healthy controls. Three of the five individual TAAs, FKBP52, PPIA, and PRDX2, showed significantly increased reactivity in breast carcinoma patients and breast carcinoma *in situ* patients compared to healthy controls. When combined into a panel, the five TAAs significantly discriminated breast carcinoma (receiver operating characteristic (ROC) area under the curve (AUC), 0.73; 95% confidence interval (CI), 0.60–0.79) and breast carcinoma *in situ* (ROC AUC, 0.80; 95% CI, 0.71–0.85) from healthy individuals. Importantly, the ROC AUC value of the panel was able to distinguish breast carcinoma *in situ*, including high grades, from healthy controls in women under the age of 50 years (ROC AUC, 0.85; 95% CI, 0.61–0.92). The authors [17] conclude that their AAb assay to a panel comprised of five TAAs allows for an accurate discrimination between early-stage breast carcinoma, especially breast carcinoma *in situ*, and healthy individuals. These results could be of interest in detecting early breast carcinoma as an aid to mammography, especially in women younger than 50 years.

## 5. Autoantibody Assay to a Panel of Tumor-Associated Antigens Comprised of MUC1, HER2, p53 and IGFBP2

Lu et al. [1] have assessed the value of an AAb assay to a panel of TAAs comprised of MUC1, HER2, p53 and IGFBP2 as a diagnostic tool for breast carcinoma. Although the serum AAb response rate to the best performing single antigen, MUC1, was no more than 20%, addition of HER2 to the panel increased the percent of positive samples to 25%, and addition of p53, and IGFBP2 increased the rate of positivity to 31% [1]. Thus, 31% of the breast carcinoma patients analyzed with an AAb assay to a panel of TAAs comprised of MUC1, HER2, p53, and IGFBP2 had serum AAbs to at least one of the four TAAs tested, suggesting that diagnostic sensitivity may be improved by using AAb assay to a panel of TAAs for detection of breast carcinoma.

## 6. Autoantibody Assay to a Panel of Tumor-Associated Antigens Comprised of p53, HER2, IGFBP-2, and TOPO2*α*

Lu et al. [1] tested the sera of 184 advanced-stage breast carcinoma patients and 134 healthy controls for AAb response to p53, HER2, IGFBP-2, and TOPO2*α*, and the responses were used to construct receiver operating characteristic (ROC) curves. They have found that response to p53 alone was not a significant predictor of breast carcinoma (area under the curve (AUC) = 0.48, *P* = .538), but combining responses to two antigens (p53 and HER2) resulted in an AUC = 0.61 (*P* = .006), and combining responses to all of the four antigens increased the AUC to 0.63 (*P* = .001). Using an algorithm weighted on logistic regression coefficients of AAb response to individual TAAs resulted in an AUC of 0.7 (*P* < .001) [1]. The authors [1] concluded that using an AAb assay to a panel of TAAs is more efficient at discriminating breast carcinoma patients from healthy controls than the use of an AAb assay to a single TAA. Nevertheless, the serum samples tested by Lu et al. [1] were obtained from patients with advanced-stage disease; whether the findings apply to patients with early-stage breast carcinoma remains to be investigated.

## 7. Autoantibody Assay to a Couplet of Tumor-Associated Antigens Comprised of Survivin and Livin

Survivin and livin are inhibitors of apoptosis protein (IAP). Yagihashi et al. [18] examined by specific ELISA using recombinant protein the prevalence of AAbs to survivin and livin in the sera of 46 breast carcinoma patients. With use of a cutoff value for positivity determined as the mean absorbance + 2 SDs for healthy controls, 11/46 (23.9%) and 15/46 (32.6%) breast carcinoma patients were seropositive for survivin and livin, respectively. The intensity of anti-survivin AAb responses did not correlate with intensity of antilivin AAb responses (*r* = 0.1628). In addition, the intensities of AAb responses to survivin or livin did not correlate with tumor size, lymph node status, or distant metastasis. However, when the AAb assay was activated to survivin and livin combined into a panel, 24/46 (52.2%) patients were seropositive for at least one of the two TAAs [18]. It has, thus, been shown that the diagnostic sensitivity may be improved by using AAb assay to a couplet of TAAs rather to a single TAA for detection of breast carcinoma.

## 8. Autoantibody Assay to a Panel of Tumor-Associated Antigens Comprised of ASB-9, SERAC1, and RELT

Zhong et al. [19] investigated by ELISA the presence of AAbs to six tumor-associated phage-expressed proteins, KLF17, COL6A1, GRWD1, ASB-9, SERAC1, and RELT in the sera from 87 breast carcinoma patients and 87 normal controls. Each of the three TAAs, ASB-9 (36/87 (41.3%), *P* = .0112), SERAC1 (41/87 (47.1%), *P* = .0009), and RELT (46/87 (52.9%), *P* = .0001) elicited significantly higher frequency of AAbs in breast carcinoma patients compared to healthy controls. When these three TAAs were combined into a panel, it was found that 70/87 (80%) breast carcinoma patients had serum AAbs to at least one of these TAAs. Measurements of AAb response to these three TAAs were combined in a logistic regression model that produced an area under the curve (AUC) equal to 0.861 in the receiver operating characteristic (ROC) curve and achieved a sensitivity of 80% and specificity of 100% in prediction of breast carcinoma (*P* = .0001). The authors [19] conclude that the use of AAb assay to a panel of TAAs, rather than to a single TAA, is a promising approach for early detection and diagnosis of breast carcinoma.

## 9. Autoantibody Assay to a Panel of Tumor-Associated Antigens Comprised of p16, p53, and c-myc

Looi et al. [20] investigated the sera from 41 breast carcinoma patients for the presence of AAbs to p16, p53, and c-myc. They found AAb to p16 in 7/41 (17.1%), AAb to p53 in 5/41 (12.2%), and AAb to c-myc in 9/41 (22%) breast carcinoma patients. This corresponded to significant increased frequency for AAbs to each of these three TAAs in breast carcinoma patients compared to healthy controls (*P* < .01). When these three TAAs were combined into a panel, it was found that 18/41 (43.9%) breast carcinoma patients had serum AAbs to at least one of these TAAs. It has, thus, been shown that with the successive addition of TAAs to the panel, there is a stepwise increase of positive AAb reaction with increased sensitivity for diagnosis of carcinoma. The authors [20] concluded that it is conceivable that AAb profiles involving different panels or arrays of TAAs might be developed in the future and the results could be useful for cancer diagnosis.

## 10. Conclusion

Breast carcinoma is the most common malignancy and the most frequent cause of death from cancer in women. Traditional diagnostic tools for early detection, namely, manual breast examination, imaging studies (mammography, ultrasound and MRI), and measurement of serum CA-15-3, are crippled with insufficient sensitivity and specificity. The mean sensitivity of mammography has been estimated to be 77% (range: 29–97%) [24–26]. The rate of false-negative mammography has been reported to be 4–34% [27, 28]. Serum AAbs to specific TAAs are detectable in cancer patients even when the tumor is obscured clinically. Evidently, the human immune system recognizes the autologous TAAs as “nonself” and makes a humoral immune response very early in the disease process. Thus, the identification of serum AAbs to TAAs could potentially be used as a novel tool for screening and early diagnosis of breast carcinoma. In a previous review published in the *Journal of Oncology*, we have focused on recent knowledge related to circulating AAbs to individual TAAs in breast carcinoma [14]. In this review, we have focused on recent knowledge related to AAb assays to tailor-made panels of TAAs in breast carcinoma. We have highlighted that measurement of AAbs to tailor-made panels of TAAs may have better promising diagnostic potential with greater sensitivity and specificity than assessment of AAbs against a single TAA. The implications of this would be that AAbs to tailor-made panels of breast carcinoma TAAs would provide a simple blood test for screening and early diagnosis of breast carcinoma. Nevertheless, it must be remembered that measurement of serum AAbs either to a single TAA or to a tailor-made panel of TAAs for screening and early diagnosis of breast carcinoma is still investigational and should be carried out along with traditional screening and diagnostic studies. Our personal viewpoints regarding the management of women having a blood test for serum AAbs to tailor-made panels of breast carcinoma TAAs are the same as for women having a blood test for serum AAbs to a single breast carcinoma TAA [14]. Presence of serum AAbs to a tailor-made panel of breast carcinoma TAAs would influence management as follows: (1) strengthen the decision to perform an immediate breast biopsy to obtain tissue for histological diagnosis rather than to wait six months for the next mammography in women with probably benign findings on current mammography (BI-RADS category 3) [29], (2) strengthen the decision to perform an immediate breast biopsy to obtain tissue for histological diagnosis rather than to wait one year for the next routine annual screening mammography in women with benign finding(s) on current mammography (BI-RADS category 2) [29], and (3) strengthen the decision to perform immediate additional imaging studies (ultrasound and/or MRI) rather than to wait one year for the next routine annual screening mammography in women with negative findings on current mammography (BI-RADS category 1) [29]. It seems that in this stage of knowledge the immediate preferred population for assaying serum AAbs to tailor-made panels of breast carcinoma TAAs would be younger women (age, <50 years) in whom mammographic screening interpretation is difficult because of dense breasts. The presence of serum AAbs to tailor-made panels of breast carcinoma TAAs in such women would hasten the assessment by additional imaging studies (ultrasound and/or MRI) and might even bring to the performance of an immediate breast biopsy to obtain tissue for histological diagnosis.

There is evidence that AAbs to breast carcinoma TAAs can be used to detect the malignancy months to years before current methods such as mammography could detect the tumor. Since AAb assays to tailor-made panels of TAAs have a better diagnostic potential than assessment of AAbs against a single TAA, it seems that AAb assays to panels of breast carcinoma TAAs have a promising future for breast carcinoma screening and early detection.

## Figures and Tables

**Table 1:** AAb assays to tailor-made panels of TAAs in breast carcinoma.

Panel of TAAs	Serum samples	Results of AAb assay	Comment	Reference
p53, c-myc, HER2, NY-ESO-1, BRCA2, and MUC1	97 IBC patients (mean age, 59 ± 12), 40 DCIS patients (mean age, 59 ± 11), and 94 controls (50 healthy blood donors and 44 women (mean age, 54 ± 11) with normal mammogram).	64% (62/97) of IBC patients and 45% (18/40) of DCIS patients were seropositive for at least one of the six TAAs. This corresponded to a sensitivity of 64% and 45% for IBC and DCIS, respectively, and specificity of 85% for both IBC and DCIS.	Positive seroreactivity was defined as an ELISA absorbance value greater than the mean + 2 SDs of the normal cohort. No correlation was found with age, tumor size, histologic grade, lymph node status, or detection method.	[3]

IMP1, p62, Koc, p53, c-MYC, cyclin B1, and survivin	64 IBC Chinese patients, 82 healthy Chinese subjects, 264 healthy USA subjects, 62 SLE USA patients, and 41 SS USA patients.	43.8% (28/64) of IBC Chinese patients were seropositive for at least one of the seven TAAs. This was significantly higher (*P* < .001) than 11% (9/82) of healthy Chinese subjects and 9.9% (26/264) of healthy USA subjects. Using classification trees based on recursive partitioning yielded a sensitivity of >70%.	Positive seroreactivity was defined as an ELISA absorbance value above the mean + 3 SDs of the healthy Chinese subjects. Only 3% (3/62) and 0% (0/41) of SLE and SS patients, respectively, were seropositive.	[15, 16]

		Discrimination between IBC patients and healthy controls gave a ROC AUC of 0.73 (95% CI, 0.60–0.79) with a sensitivity of 55.2%, a specificity of 87.9%, and a diagnostic accuracy of 75.1%.	Definition of positive seroreactivity was not reported. Except for age of DCIS patients, no correlation was found with histologic type and grade, tumor size, lymph node status, and absence or presence of ER, PR and HER-2.	
PPIA, PRDX2, FKBP52, HSP-60, and MUC1	60 early-stage IBC patients, 82 DCIS patients, and 93 matched healthy controls (mean age, 55).	Discrimination between DCIS patients of all ages and healthy controls gave a ROC AUC of 0.80 (95% CI, 0.71–0.85) with a sensitivity of 72.2%, a specificity of 72.6%, and a diagnostic accuracy of 72.4%.		[17]
		Discrimination DCIS patients under 50 years of age and healthy controls gave a ROC AUC of 0.85 (95% CI, 0.61–0.92) with a sensitivity of 73.6%, a specificity of 81.6%, a diagnostic accuracy of 73.4%, a predictive positive value of 67.3%, and a negative predictive value of 85.7%.		

MUC1, HER2, p53, and IGFBP2	Number of patients and controls was not reported. 18% of patients had early-stage IBC and 82% had late-stage IBC. Age range, 18–75 years. Controls were age- and gender-matched.	31% of IBC patients were seropositive for at least one of the four TAAs. Seropositive rate for healthy controls was not reported.	Positive seroreactivity was defined as an ELISA absorbance value greater than the mean + 3 SDs of the normal cohort.	[1]

p53, HER2, IGFBP-2, and TOPO2*α*	184 late-stage IBC patients (most of them received prior treatment) and 134 healthy controls.	Discrimination between IBC patients and healthy controls gave a ROC AUC of 0.63 (*P* < .001). Using an algorithm weighted on logistic regression coefficients resulted in an AUC of 0.7 (*P* < .001).	Positive seroreactivity was defined as an ELISA absorbance value greater than the mean + 3 SDs of the normal cohort.	[1]

Survivin and livin	46 IBC patients and 10 healthy controls (blood donors).	52.2% (24/46) of IBC patients were seropositive for at least one of the two TAAs. Seropositive rate for healthy controls was not reported; however, the difference between IBC patients and healthy controls was reported to be statistically significant (*P* < .05).	Positive seroreactivity was defined as an ELISA absorbance value greater than the mean + 2 SDs of the normal cohort. No correlation was found with tumor size, lymph node status or distant metastasis.	[18]

ASB-9, SERAC1, and RELT	87 IBC patients and 87 normal controls.	80.4% (70/87) of IBC patients were seropositive for at least one of the three TAAs. Discrimination between IBC patients and healthy controls gave a ROC AUC of 0.861 with a sensitivity of 80% and a specificity of 100% (*P* = .0001).	Definition of positive seroreactivity was not reported. AAbs were measured by phage protein ELISAs. A logistic regression model and leave-one-out validation was used to evaluate predictive accuracies.	[19]

p16, p53, and c-myc	41 IBC patients and 82 normal controls.	43.9% (18/41) of IBC patients and 2.4% (2/82) of normal controls were seropositive for at least one of the three TAAs (*P* < .01). This corresponded to a sensitivity of 43.9%, specificity of 97.6%, false negative rate of 56.1%, false positive rate of 2.4%, positive predictive value of 90%, and negative predictive value of 77.7%.	Positive seroreactivity was defined as an ELISA absorbance value greater than the mean + 3 SDs of the normal cohort.	[20]

AAb: autoantibody; TAA: tumor-associated antigen; IBC: invasive breast carcinoma; DCIS: ductal carcinoma in situ; SD: standard deviation; SLE: systemic lupus erythmatosus; SS: Sjogren's syndrome; ROC: receiver operating characteristic curve; AUC: area under curve; CI: confidence interval; ER: estrogen receptor; PR: progesterone receptor.
